# Improved performance of Bis-GMA dental composites reinforced with surface-modified PAN nanofibers

**DOI:** 10.1007/s10856-021-06557-z

**Published:** 2021-06-30

**Authors:** Parisa Amiri, Zahra Talebi, Dariush Semnani, Rouhollah Bagheri, Hossein Fashandi

**Affiliations:** 1 Department of Textile Engineering, Isfahan University of Technology, Isfahan, 84156-83111 Iran; 2 Department of Chemical Engineering, Isfahan University of Technology, Isfahan, 84156-83111 Iran

## Abstract

In the present work, polyacrylonitrile (PAN) nanofibers reinforced dental composites were investigated to achieve the improved interfacial adhesion between the PAN nanofiber and resin matrix using surface modification of nanofibers. PAN nanofibers mat were prepared by electrospinning and then, surface treated with the activated bisphenol A glycidyl methacrylate (Bis-GMA)/triethyleneglycol dimethacrylate (TEGDMA) (50/50 mass ratio) dental resin followed by photo-curing. Also, the treated nanofibers mat was milled into a powder to achieve the uniform distribution of nanofibers in the matrix resin. The reinforced dental composite were prepared by mixing the various mass fraction of the powder (0.5–15 wt%) with the Bis-GMA/TEGDMA dental monomers. The effect of weight ratio of surface-modified nanofibers to blend resin on the chemical structure, morphology, compression and flexural properties, color and polymerization shrinkage of dental composites was evaluated. The results showed that using surface-treated nanofibers with content of 5 wt% enhanced the compression strength, flexural strength, flexural modulus and work of rupture of the resultant dental composite by factors of 23%, 7%, 80%, and 145%, respectively, comparing to the unreinforced neat resin. Also, the polymerization shrinkage reduces by 37%. These significant improved properties of the dental composite could be due to the semi-interpenetration network formation between surface-modified nanofibers and resin matrix and well distribution of nanofibers in the dental resin. Further increasing the nanofiber content led to poor mechanical properties of obtained dental composites. The results also, revealed that the color of resin composite could be whiter using modified PAN nanofibers as the filler.

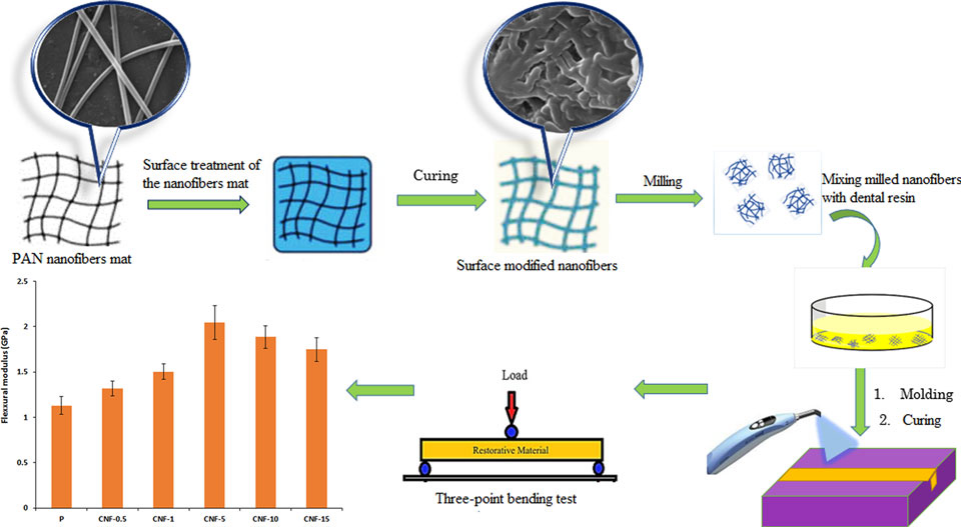

## Introduction

In recent years, dental composites have been attracted great attention for restorative dentistry. Having fascinating appearance, better clinical handling, and less unfavorable side effects, these composites rapidly replaced the traditional materials such as amalgam, particularly in the restoration of incisor teeth [1–3].

The blend of BIS-GMA and TEGDEMA monomers has been widely used as the most popular dental materials [4–6]. Mechanical properties are the most important issue in dental composites. The fracture plays a vital role in the mechanical failure which is affected by the interfacial adhesion between filler and resin matrix [7]. Researchers have widely focused on the filler content, particle size, enhancing adhesion between filler and resin which improve the physical and mechanical properties of dental composites [8–10]. It has been demonstrated that the mechanical properties of dental composites are severely affected by size and mass of the filler particles. Many attempts have been made to gradually reduce the filer particle size from micron to nanometer to achieve considerable improved mechanical properties of the dental composites [11–13].

The interfacial adhesion between filler and matrix profoundly affected the mechanical properties of the dental composite [14–16]. In this regard, surface properties of fillers play the pivotal role. Fillers with a high specific surface area provide higher interaction between matrix and filler and interpenetration network (IPN) structure of the filler and matrix is formed. Under these conditions, strong interfacial bonds are created between different phases, which could account for the enhanced mechanical properties of the dental composite [17, 18].

Liu et al. modified the surface of hydroxyapatite whisker with poly Bisphenol A glycidyl methacrylate (Bis-GMA) to achieve enhanced adhesion between the whisker and matrix. Using the modified whisker as filler increased the bending strength and reduced the contraction of the dental composite compared to the unmodified whisker. Poly(Bis-GMA) on the surface of whisker acted as a layer by which the interfacial compatibility and hence, the interaction between modified whisker and matrix enhances. Consequently, the modified whisker was uniformly distributed within the resin and the polymerization shrinkage of obtained composites was considerably decreased. Totally, graft polymerization on the filler surface was a promising technique to prepare dental composites [19, 20].

The reinforcement of triethyleneglycol dimethacrylate (TEGDMA)/GMA dental resin with poly(Bis-GMA)-grafted silanized hydroxyapatite whisker (PGSHW) has been reported by Liu et al. They found that S-SiO_2_ remarkably increased the degree of resin conversion. In additiona, The combination of PGSHW:S-SiO_2_ with mass ratio of 2:4 enhanced the bending strength, bending modulus, compression strength and work of rupture of the composite by 39.1%, 61.1%, 50.1%, and 85.9%, respectively, compared to the composite containing PGSHW [20, 21].

The effect of fiber surface modification of the dental composite reinforced with ultra-high molecular weight polyethylene fiber was evaluated by Bahramian et al. Their results revealed that corona and silane surface modification of fibers increased the surface roughness of fibers leading to the better interfacial adhesion between the fibers and matrix resin. Therefore, flexural properties and fracture toughness of the reinforced composite with surface-modified fibers significantly improved [22].

Yi et al. found that the tight interfacial adhesion between the glass fiber and Bis-GMA/TEGDMA dental resin was achieved by surface coating the glass fiber with poly(methyl methacrylate) (PMMA) and polydopamine (PDA) especially in the PDA/PMMA coated fibers. Subsequently, the flexural strength and modulus of the resulting reinforced dental composite increased [23].

In recent years, many works have been done to reinforce dental resins with electrospun nanofibers such as nylon 6, PAN, and nanoscale glass fibers. The nanometric-sized diameter of fibers provides a high specific surface area which improves the interfacial area between the fibers and dental resin leading to improved mechanical properties of nanofiber reinforced dental composites [24–26]. Moreover, the nanofibers hinder the growth of a crack initiated in the matrix dental resin under applied stresses and hence, the dental resin is reinforced and improved mechanical properties are achieved [27]. However, it was found that the improved mechanical properties of nanofibers reinforced dental composites depended on the wettability of the nanofibers by the matrix resin which was affected by nanofibers surface properties and the resin viscosity [28].

Considering the great contribution of nanofiber surface properties and enhancing interfacial adhesion between fibers and matrix resin to the mechanical properties of dental composites, this work is aimed at developing a new Bis-GMA based dental composite reinforced with surface treated PAN nanofibers mat with the activated Bis-GMA/TEGDMA dental resin followed by photo-curing. As the reinforcement of the dental resin with electrospun nanofibers mat or felt via layer by layer impregnation with dental resin is not applicable method for the dentistry, the treated nanofibers mat was milled into a powder to achieve the uniform distribution of nanofibers in the matrix resin. The reinforced dental composite finally was prepared by mixing the various mass fraction of powder with the Bis-GMA/TEGDMA dental monomers. The effect of weight ratio of surface-treated nanofibers to blend resin on the dental composite properties was evaluated via chemical structure, morphology observation, compression and flexural properties, color and polymerization shrinkage of composites.

## Experimental

### Materials

Polyacrylonitrile (PAN) powder (Mw = 150000 g/mol) and N,N-dimethylformamide (DMF) solvent were purchased from Merck CO. for production of the electrospun nanofibers. Bis-GMA and TEGDMA monomers, camphorquinone as photo-initiator, and 2-(dimethylamino) ethyl methacrylate (DMAEMA) as co-initiator were also supplied from Merck CO.

### Electrospinning of PAN nanofibers

The solution of 13 wt% PAN dissolved in DMF was prepared. The electrospinning of PAN nanofibers was carried out by a custom-made electrospinning apparatus used a high voltage power supply and a syringe as feeder system. The solution was fed to the syringe pump at 8.5 ml/min flow rate. The nanofibers were collected on the aluminum foil sheet at the collector drum rate of 5 rpm. The applied voltage was kept at 9.6 kV, and the distance between the needle tip and the collector was 16 cm.

### Surface modification on PAN nanofibers

A coating mixture of Bis-GMA/TEGDMA (50/50 weight ratio), 0.5 wt% QC and 1 wt% DMAEMA were prepared. The PAN nanofibers mat was removed from the aluminum foil and immersed into the coating mixture at 2 wt% of mixture and then light-cured by LED lamp. The treated PAN nanofibers mat was cryogenically fractured in liquid nitrogen and milled into a powder with particle size range of 500–600 μm which used as the filler of reinforced resin composite samples.

### Dental composite preparation

Dental resin mixtures were prepared by mixing the Bis-GMA and TEGDMA monomers with QC as photo-initiator DMAEMA as co-initiator at room temperature. The composition of resin mixtures are described in Table 1. The nanofiber powder added to the resin mixtures at weight ratios in the range of 0.5–15 wt% according to Table 1. Then dental resins mixed thoroughly and were placed in the dark vacuum chamber for 3 h at 24 °C to remove the air bubbles in the resin mixtures. The dental resins then cured by two exposures of 40 s using a dental curing LED lamp.

**Table 1:** Composition of the dental composite samples reinforced with PAN surface-modified nanofibers

Sample code	CNF-0.5	CNF-1	CNF-5	CNF-10	CNF-15
Bis-GMA (g)	1/2	1/2	1/2	1/2	1/2
TEGDMA (g)	1/2	1/2	1/2	1/2	1/2
Nanofibers (g)	0.012	0.024	0.12	0.24	0.36
CQ (g)	0.012	0.012	0.012	0.012	0.012
DMAEMA (g)	0.024	0.024	0.024	0.024	0.024

### Characterization

The morphology of PAN electrospun nanofibers mat, ground modified nanofibers as the filler of dental resin composites and the fracture surface of the composite samples after three-point bending test were evaluated by scanning electron microscopy (SEM, VEGA\\TESCAN).

A spectrometer (FTIR, Hartmann & Braun, MB-Series 100, Canada) was used to evaluate the chemical groups of the samples.

### Mechanical properties

The flexural properties of resin composites were examined in a three-point bending test according to the ISO4049 standard using a Hounsfield universal test machine with a 16 mm span and crosshead speed of 0.5 mm/min. The sizes of composite specimens were 2 × 2 × 25 mm for this test and light-cured by four exposures of 40 s by LED lamp. Flexural strength (FS) and flexural modulus (FM) were calculated from Eqs. (1) and (2).

1 $${FS}= \frac{3FL}{2{bh}^{2}}$$

2 $${{FM}} = \frac{{{{mL}}^3}}{{4{{bh}}^3}}$$

where *F* is the maximum applied load (N), the *L* is the spam length (16 mm), *b* is the width of the test sample (mm) and *h* is the thickness of the test sample (mm) and *m* is the slope of the linear portion of load-deflection curve.

The compressive strength of modified nanofibers reinforced composites was determined according to the ISO9917-1 standard by a Hounsfield universal test machine with speed of 0.5 mm/min. The cylindrical sample test (*D* = 4 mm, *H* = 6 mm) prepared for the compressive test.

The linear polymerization shrinkage of resin composites calculated from the change percent of sample height before and after light-curing. A laser detector machine was used to measure the height of test specimens. The test sample sizes were the same as those of the compressive test.

The color change of the dental composite samples during the photo-curing examined by the evaluation of their whiteness and yellowness indexes. The whiteness and yellowness indexes were measured by a reflectance spectrophotometer (Tex Flash). The disc shape test samples (*D* = 9, H = 4.5 mm) were prepared and photo-cured in the different light-curing of 5, 10, 20, 40, and 60 s.

### Statistical analysis

The mechanical properties of the dental composites reinforced with the surfaced-modified nanofibers have been also investigated and compared to those of the neat resin. Statistical analysis of ANOVA was used to analyze the results. A *P* value of < 0.05 was assumed to be significant.

## Results

**Fig. 1:**
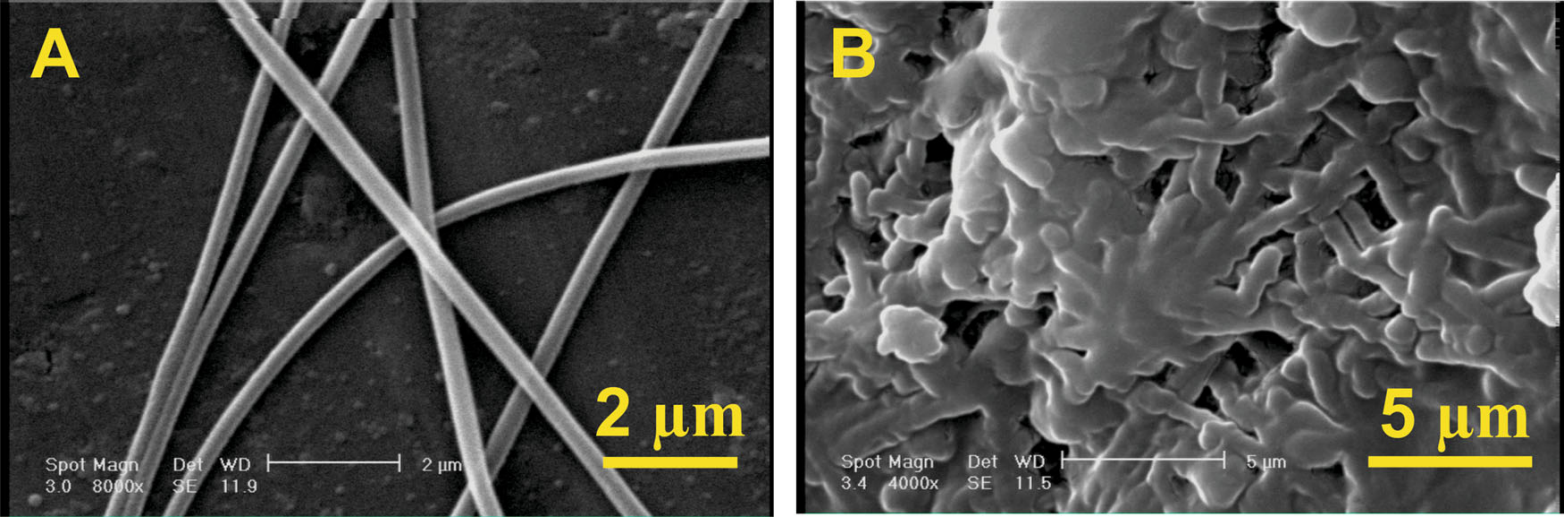
SEM images of electrospun PAN nanofibers (**A**), electrospun PAN nanofibers coated with Bis-GMA/TEGDMA resin (**B**)

Fig. 1A shows the SEM image of the electrospun PAN nanofibers before coating. As shown in Fig. 1, bead-free PAN nanofibers with smooth surface have been successfully produced. The diameter of the produced nanofibers ranges between 270–580 nm with the average value of 387 nm. The SEM image of surface-modified nanofibers with Bis-GMA/TEGDMA resin after grinding is also shown in Fig. 1B by which can clearly confirm reveals an apparent that the resin has been coated on the surface of nanofibers. The average diameter of the coated nanofibers was measured to be 675 nm, which is obviously larger than that of the initial nanofibers.

### Chemical characterization

**Fig. 2:**
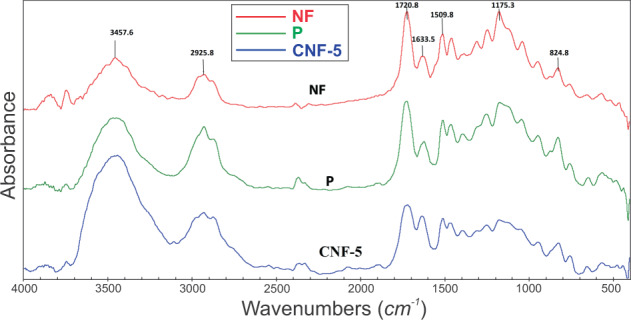
FTIR spectra of the neat resin (P), the surface-modified nanofibers (NF) and the dental composite reinforced with surface-modified nanofibers (CNF-5)

FTIR spectra of the neat resin, surface-modified nanofibers with resin and the dental composite reinforced with the surface-modified nanofibers are shown in Fig. 2. The sharp peak at 1720 cm^−1^ is assigned to the stretching vibrations of C=O bonds. The stretching vibrations of C-O bonds can be observed as a weak peak at 1110 cm^−1^. The peak at 3445 cm^−1^ is attributed to O-H stretching vibrations. Two peaks at 1625 and 1510 cm^−1^ are due to aromatic rings of Bis-GMA. The stretching and bending vibrations of C-H bonds in CH_2_ groups are observed in 2955 and 1435 cm^−1^, respectively. The results indicate that no chemical interaction has occurred between the surface-modified nanofibers and resin matrix.

### Mechanical properties

#### Compressive strength

**Fig. 3:**
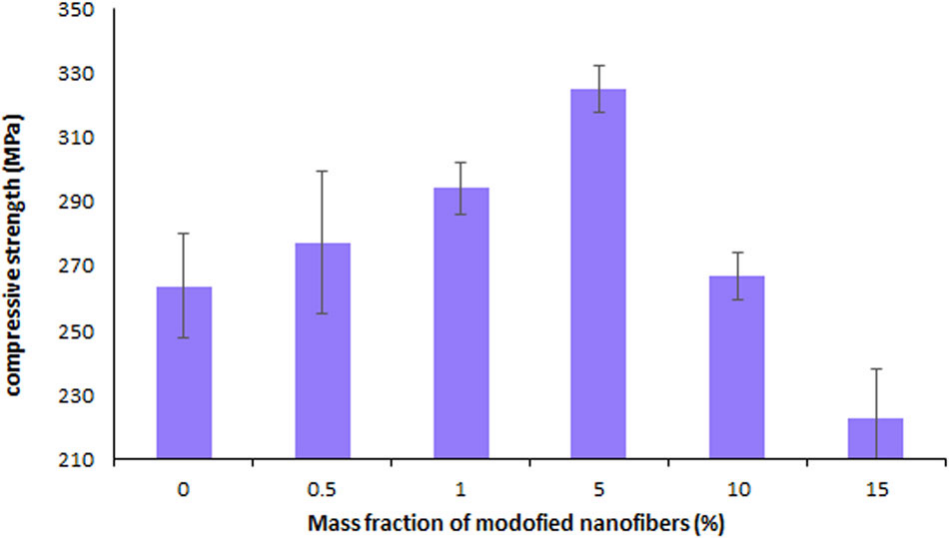
Compressive strength of the dental composite containing different contents of surface-modified PAN nanofibers

Figure 3 shows the compressive strength of the dental composites filled with different contents of surface-modified PAN nanofibers. The compressive strength of the control sample (pristine Bis-GMA/TEGDMA resin), can be also deduced from Fig. 3. The results demonstrate the incorporation of the 0.5–5 wt% surface-modified PAN nanofibers into the neat resin increases its compressive strength. Using 5 wt% nanofibers, the compressive strength of the dental composite is significantly improved by 23% comparing to the unreinforced neat resin.

Further increasing the nanofiber content of composite to 10 and 15 wt% leads to poor compressive strength. The ANOVA statistical analysis confirms the significant differences between the obtained results.

#### Flexural properties

**Fig. 4:**
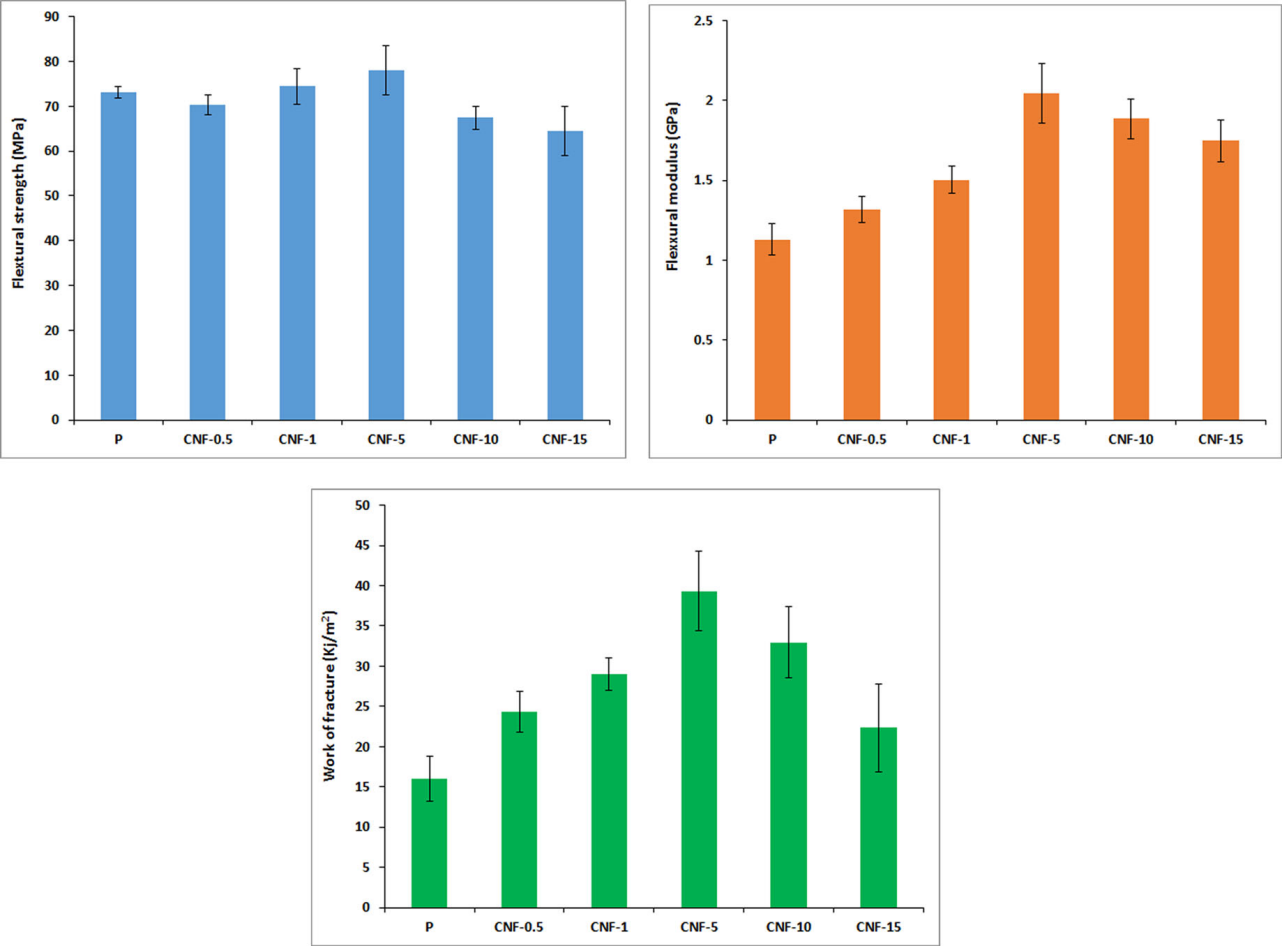
Flexural properties of the dental composite containing different contents of surface-modified PAN nanofiber

The FS, FM, and WOF of the resin composites as a function of modified nanofiber content are shown in Fig. 4. As can be seen, flexural properties of the resin composite containing the surface-modified nanofibers with maximum content of 5 wt% by have been improved compared to the neat resin. FS, FM, and WOF of the neat resin are 73.11 ± 1.28 MPa, 1.13 ± 0.10 GPa, and 16.03 ± 2.13 kJ/m^2^, respectively. In the resin composite reinforced with 5 wt% surface-modified nanofibers, the values of FS, FM, and WOF have significantly increased by 7%, 80%, and 145%, respectively, compared to the unreinforced resin. ANOVA statistical analysis also confirms the results.

### Surface fractural morphology of composites

**Fig. 5:**
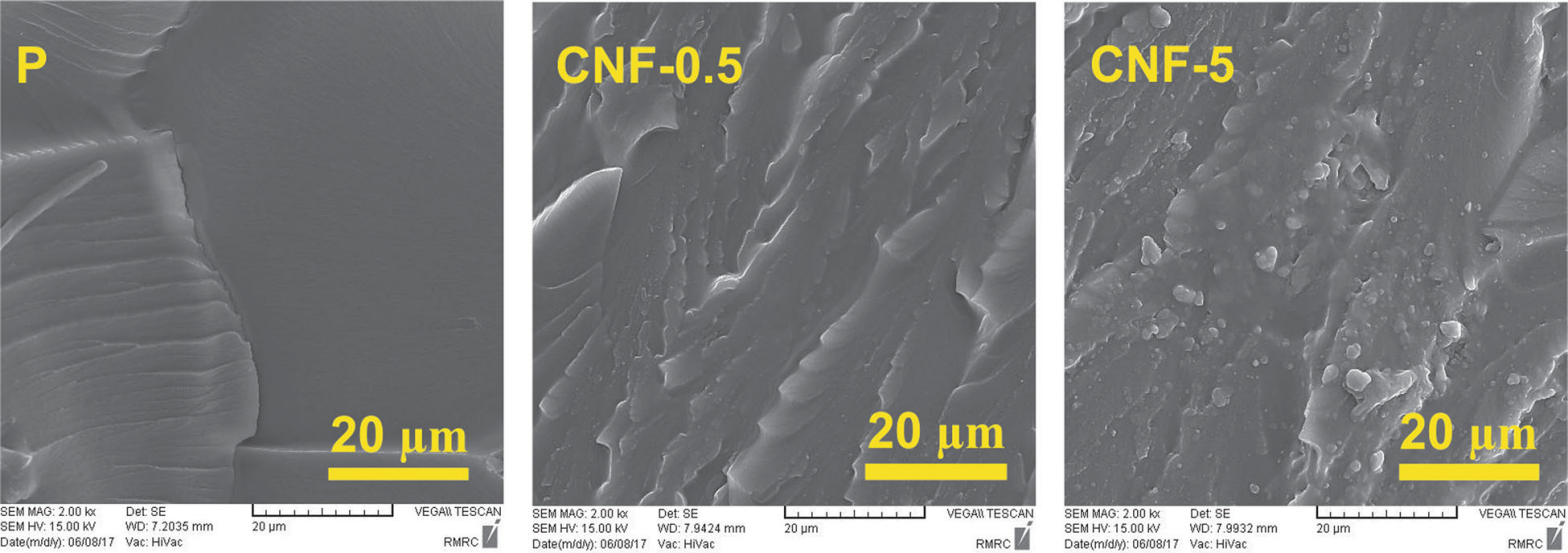
SEM images of the fractured surfaces of dental composite samples

Fracture surface images of the neat resin and the composites reinforced with the surface-modified nanofibers are shown in Fig. 5. As shown, the fractured surface of the neat resin is smooth while, the composites reinforced with the surface-modified nanofibers exhibit a very rough fractured surface.

### Polymerization shrinkage

The polymerization shrinkage of the neat resin and the composite reinforced with 5 wt% surface-modified nanofibers are reported in Table 2. The results showed that polymerization shrinkage significantly decreases by 37% for the reinforced composite in comparison to the neat resin.

**Table 2:** The polymerization shrinkage of the neat resin and the composite reinforced with 5 wt% surface-modified nanofibers

Sample	CNF-5	*P*
Average linear polymerization shrinkage (%)	3.3 ± 0.63	5.29 ± 0.41

### Resin composite color

**Fig. 6:**
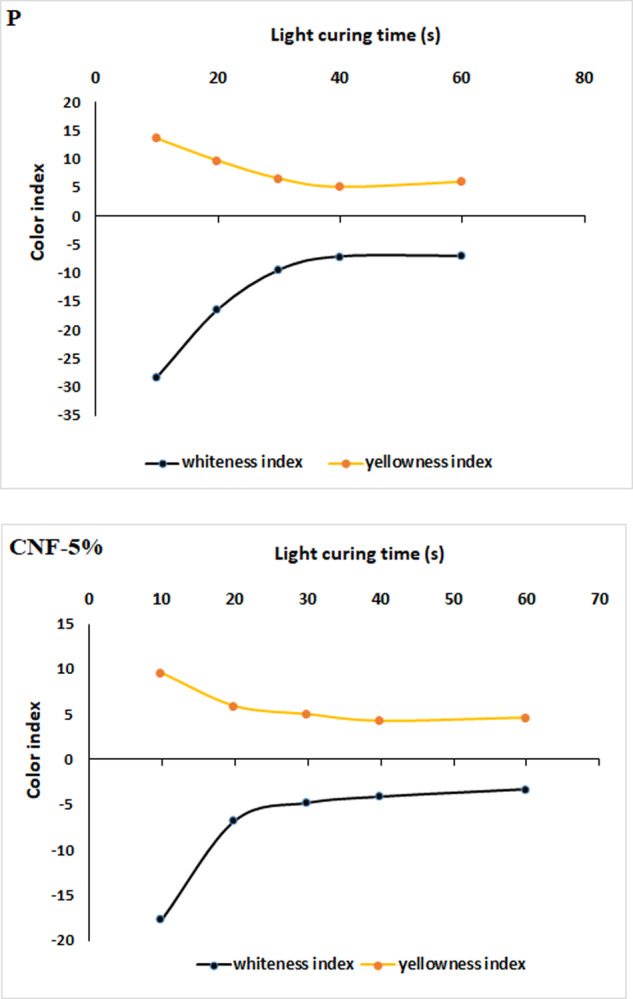
Whiteness and yellowness indexes of (**a**) the neat resin and (**b**) the composite reinforced with 5 wt% surface-modified nanofibers

The effect of composite light-curing time on the whiteness and yellowness indexes of the neat resin and the composite reinforced with 5 wt.% surface-modified nanofiber are displayed in Fig. 6. In both samples, whiteness index increases and yellowness index decreases by increasing the light-curing time to 40 s. These indexes are not noticeably changed after 40 s. The results demonstrate that yellow color of resin composite due to the QC as the photo-initiator changed to white color by photo-polymerization. Moreover, the yellowness index of the composite reinforced with nanofibers (4.3) is lower than that of the neat resin (5.1). These results reveal that the color of resin composite could be whiter using modified nanofibers as filler.

## Discussion

Excellent mechanical properties and low polymerization shrinkage are needed to achieve the improved performance dental composites. The interfacial adhesion between filler and resin matrix is the main factor that rigorously influenced the mechanical properties of dental composites. In this regard, the surface modification of filler is the most efficient method used to enhance the adhesion between the filler and matrix.

In this work, the PAN nanofibers reinforced dental composites were evaluated in which the surface modification of PAN nanofibers mat by coating with the activated Bis-GMA/(TEGDMA) dental resin was carried out to improve the dental composite performance. The results show the incorporation of the 0.5–5 wt% surface-modified PAN nanofibers into the neat resin increases its compressive strength and flexural properties. In fact, the nanofibers surface modification used to increase the adhesion of PAN nanofibers to resin leading to further improvement of mechanical properties of the PAN nanofibers reinforced composites. This can be attributed to the formation of semi-IPN between the coated polymer on the surface of PAN nanofibers and Bis-GMA/TEGDMA matric resin in light-cured composites [18]. The coated polymer on the surface of nanofibers can partly dissolve in the matrix resin monomers via the methacryloyl groups interactions leading to the entanglement of the coated polymer chains with the resin network after light curing. Therefore, better interfacial adhesion to the matrix resin is obtained for the surface-modified PAN nanofibers. Hence, an impressive stress transfer from the resin to the filler and improved mechanical properties of the final composite have occurred. In another research by Lia et al. Bis-GMA/TEGDMA monomers were grafted on the surface of hydroxylapatite whiskers as a filler of Bis-GMA/TEGDMA dental resin composite which improved the compressive strength of obtained composite [19, 21, 29].

However, by further increasing the content of surface-modified nanofibers to values of 10 and 15 wt%, not only no improvement in the mechanical properties of the resin composite can be observed, but also a decrease in the mechanical properties to values less than that of the neat resin has occurred. In fact, at reinforced nanofiber weight ratios >10%, poor dispersion of filler might have occurred which reduces the interfacial adhesion between nanofiber and matrix resin leading to the uneven stress transfer from matrix to the nanofibers as a filler and hence, decreased compressive and flexural properties are resulted [25].

The high roughness of the fractured surface of composite indicates its high resistance to the applied force. Energy dissipation during the fracture process could also be increased due to nanofiber crack bridging and deflection by nanofibers. Surface modification of nanofibers by coating with Bis-GMA/TEGDMA resin enhances the interfacial adhesion between nanofiber as a filler and matrix resin via the IPN structure formation leading to better stress transfer. Therefore, the resistance of the reinforced resin composites to fracture is increased [23, 30].

The interaction of the filler and matrix resin plays the pivotal role in the polymerization shrinkage of dental composites. It is expected that the polymerization shrinkage could be decreased by increasing the filler/resin interface [31, 32]. Therefore, the results clearly reveal that the polymerization shrinkage of reinforced dental composites with treated nanofibers decreases by 37% compared to the unreinforced neat resin due to good interface adhesion between the nanofibers and resin matrix.

## Conclusion

Dental composites reinforced with surface-treated polyacrylonitrile (PAN) nanofibers were investigated. Surface modification of the produced nanofibers mat was carried out by coating the nanofibers mat with the activated Bis-GMA/(TEGDMA) dental resin followed by photo-curing. Also, the treated nanofibers mat was milled into a powder to achieve the uniform distribution of nanofibers in the matrix resin.

The electrospun nanofibers showed an average diameter of 387 nm, which increased to 675 nm after surface treatment. Surface treatment increased the adhesion of PAN nanofibers to resin consequently, enhanced the mechanical properties due to the interpenetration network formation between surface-modified nanofibers and matrix. Also, the well distribution of the PAN nanofibers in the dental resin led to further improvement of mechanical properties of the PAN nanofibers reinforced composites. Effect of weight ratio of surface-modified nanofibers to blend resin on the chemical structure, morphology observation, compression and flexural properties, color, and polymerization shrinkage of reinforced composites were investigated. The result showed that the incorporation of the 0.5–5 wt% surface-modified PAN nanofibers into the neat resin increased its compressive strength and flexural properties. The compression strength, flexural strength, flexural modulus and work of rupture of 5 wt% nanofibers reinforced composite enhanced by factors of 23, 7, 80, and 145%, respectively, comparing to the unreinforced neat resin. Further increasing the nanofiber content of composite led to poor mechanical properties of composite. Also, the polymerization shrinkage reduces by 37% and the color of resin composite could be whiter using modified PAN nanofibers as the filler.
